# A Flexible Strain Sensor Based on a Conductive Polymer Composite for *in situ* Measurement of Parachute Canopy Deformation

**DOI:** 10.3390/s100908291

**Published:** 2010-09-02

**Authors:** Cédric Cochrane, Maryline Lewandowski, Vladan Koncar

**Affiliations:** 1 University Lille Nord de France, F-59000 Lille, France; 2 ENSAIT, GEMTEX, F-59100 Roubaix, France

**Keywords:** carbon black, conductive polymer composite, flexible sensor, textile strain gauge

## Abstract

A sensor based on a Conductive Polymer Composite (CPC), fully compatible with a textile substrate and its general properties, has been developed in our laboratory, and its electromechanical characterization is presented herein. In particular the effects of strain rate (from 10 to 1,000 mm/min) and of repeated elongation cycles on the sensor behaviour are investigated. The results show that strain rate seems to have little influence on sensor response. When submitted to repeated tensile cycles, the CPC sensor is able to detect accurately fabric deformations over each whole cycle, taking into account the mechanical behaviour of the textile substrate. Complementary information is given concerning the non-effect of aging on the global resistivity of the CPC sensor. Finally, our sensor was tested on a parachute canopy during a real drop test: the canopy fabric deformation during the critical inflation phase was successfully measured, and was found to be less than 9%.

## 1. Introduction

To satisfy an increasing demand in the field of innovative and smart materials, intelligent textile structures have to be imagined and designed. The need for sensors and actuators is an important issue for automotive, aeronautic and sport application areas. Traditional sensors (strain gauges, temperature probes…) are neither flexible, nor compatible with deformations of textile fibres and fabrics, even if in some cases, they can be used on flexible structures with specific mounting schemes. For instance, in the late seventies, Heinrich used the “Omega structure” to evaluate deformations of a parachute canopy in a wind tunnel [1,2]. More recent developments in sensing adapted to textile structures consisted in integrating metallic yarns (stainless steel mainly) or optical fibres (Fibre Bragg Grating principle) [3–5]. Another way to develop flexible (*i.e.*, compatible with textile behaviour) mechanical sensors (electrosensitive to elongation or pressure) is to use electro-conductive materials based on conductive polymer composites (CPC). Many works are related to the use of carbon black [6–8], short carbon fibres [9] or carbon nanotubes [10] as fillers in these CPC sensors. In addition to electromechanical applications, these materials can be used as chemical sensors (solvent detection or humidity) [11–13] or temperature probes [14].

Often, CPC materials are prepared by melt mixing a conductive filler within a thermoplastic matrix. Commercial products based on latex or silicone loaded with conductive fillers and obtained by drying and/or cross linking can also be found. In our previous work [15], the possibility of using a CPC solution, based on a thermoplastic elastomer and carbon black particles, to realize a strain sensor on a light fabric has been demonstrated. In this article, the results related to the optimization of the operating procedure, the effects of aging, electromechanical cyclic measurement and strain rate on the electrical resistivity of the sensor are revealed. Moreover, the performance of our sensor is demonstrated in a real parachute drop test, in which the deformation of the parachute canopy during the inflation phase is successfully measured. These flight tests were performed in collaboration with our partner, the CEV (*Centre d’essai en vol*, Centre for Flight Tests) based in Toulouse, France. The materials and procedure to make the CPC sensor are discussed only briefly here, since all the details can be found in [15].

## 2. Materials

### 2.1. CPC materials

Evoprene 007 from Alphagary was used as polymer matrix. It belongs to the class of thermoplastic elastomers (TPE) and was found to contain a large amount of inorganic filler, identified as calcium carbonate by thermogravimetric analysis [16]. This filled Styrene-Butadiene-Styrene co-polymer has a density equal to 1.16 g.cm^−3^. The conductive filler particles are made of highly structured carbon black (CB) provided by Evonik (Degussa Corp.). The average particle diameter is 18 nm, but the SEM observation has revealed the presence of aggregates with a size of up to 200 nm [16]. A CPC solution is prepared by solvent mixing using a standard grade of chloroform, with a density of 1.49 g.cm^−3^.

### 2.2. Other materials

The CPC strain sensor was deposited on a lightweight and thin polyamide 6.6 (Nylon 6.6) fabric. This fabric, used as parachute canopy, has a basis weight of 42 g.m^−2^, and a thickness of 45 μm. In all experimental tests, the fabric sample dimensions are 300 mm × 50 mm cut in the weft (perpendicular to the fabric production) direction.

The electromechanical sensor is a conductive track obtained by scraping a little amount of CPC solution on a mask formed by a rectangular die (2.5 mm × 100 mm) cut in a steel sheet. Five different masks with thicknesses of 70 μm, 100 μm, 120 μm, 150 μm and 200 μm (±5%) were used. The main change, compared to our previous work [15], is that the die is now cut by laser beam. This clean cut gives a precise die and helps to improve the reproducibility of samples. A new coating table based on electromagnets has been developed in order to support firmly the steel mask on the Nylon 6.6 fabric during the coating, also helping to improve sensor quality. The table surface is perfectly flat and protected with a silicone layer.

Electrical connections are realized using stainless steel yarns from Bekintex, composed of 2 × 275 continuous filaments of 12 μm. The electrical contacts between the sensor and yarns are sealed by means of a small drop of CPC solution.

The CPC coating integrated on the fabric must be protected against abrasion. An acrylic latex (Appretan 96100) provided by Clariant which upon drying forms a thin protective film is sprayed on the conductive track.

## 3. Experimental

### 3.1. Integration of sensor on parachute canopy fabric

Samples of fabric are preliminarily washed with acetone and ironed to remove all creases. Then, they are placed on the electromagnetic table. It is important to remove all air bubbles between the table and the fabric. The steel mask is put on the fabric, the longer direction of the rectangular die placed perfectly parallel to the weft yarns. Then the electromagnetic table is activated to maintain the mask and fabric solidly in place.

A CPC solution obtained by solvent mixing at low temperature was prepared with the following concentrations: 35 wt.−% of CB, 65 wt. −% of Evoprene, and 6 wt. part of chloroform per part dry CPC. A small amount of this CPC solution is deposited at one end of the mask and scraped with a blade over the length of the die to the other end. After coating, the CPC was completely dry in approximately one minute. The last steps consist in adding the electrical stainless steel wire connections and depositing the protective latex layer on the CPC track. The effective length of the sensor is equal to the distance between the stainless steel wires (80 mm in this study).

### 3.2. Thickness measurement

Thickness of the conductive track (without latex layer) is measured with an optical profilometer (Cotec Altisurf 500). The coated fabric is stuck on a clean glass slide and placed under the measuring head. A scan of the sample is performed to record its surface roughness, from which is deduced the thickness of the CPC sensor. Thickness values used in this paper are an average of at least 20 profiles measured along the track.

### 3.3. Electrical measurement

This section describes the experimental methods used to measure the effect of different parameters such as CPC thickness, aging, and strain rate on the electrical response of the sensors.

In static measurements of the electrical resistance of the CPC sensor, a multimeter (Keithley 617) interfaced with a computer is used to record and process data. A voltage *V* is applied to the sensor and the current intensity *I* is measured. The applied voltage ranged from 0 to 10 V, with an automatic increment of 0.1 V. For each sample, the *I*/*V* curve is plotted and the electrical resistance *R* (Ω) is deduced from the slope of the curve. The electrical resistivity of the CPC sensor *ρ* (Ω.m) is then calculated from *R* and the dimensions of the track (Equation 1):

(1) R=(ρ×L)/(l×e)

with *R*, the electrical resistance (Ω); *ρ*, the CPC electrical resistivity (Ω.m); *L*, the length of the track (80 × 10^−3^ m); *l*, the width of the track (2.5 × 10^−3^ m) and *e*, the thickness of the track (m).

In order to compare sensors with slight variations in their intrinsic resistance value, a relative electrical resistance is defined as follows (Equation 2):

(2) $$Rr=(R-R_{0})/R_{0}$$

where *R* and *R_0_* are the electrical resistance (Ω) of the sensor during measurement and its initial value, respectively. The sensor results will therefore be discussed preferentially in terms of this parameter.

To record the electrical resistance during dynamic tests (tensile tests) a data acquisition card (KUSB 3100) from Keithley is used. This card can measure voltages ranging from −10 V to 10 V with a frequency up to 50 kHz. A simple bridge is used to measure sensor resistance. Equation 3 defines the electrical resistance of the sensor in this set-up:

(3) R=V×Rb/(Vi−V)

where *V* is measured voltage (V), *Rb* bridge resistance (Ω), *Vi* voltage of power supply (V), and *R* is the electrical resistance of sensor (Ω).

#### (a) Aging effect measurement

In order to evaluate the stability of the sensor, the electrical resistance of two control sensors (named A and B) has been monitored over a long period of time. During 170 days, the fabric samples equipped with a CPC sensor were left in a horizontal position, without any external strain, under ambient laboratory conditions (*i.e.*, without humidity or temperature control). Every 2 or 3 days, the electrical resistance, ambient humidity and temperature were recorded.

#### (b) Influence of the strain rate

Sensors were calibrated under different elongation rates in order to investigate the influence of this parameter on sensor behaviour. Dimensions of the fabric test sample were 200 mm × 50 mm, and the sensor track was placed at the centre of the sample. A conventional tensile testing machine (MTS 2/M) allowing sample extensions up to 1,000 mm/min has been used. Seven different elongation rates were studied: 10 mm/min, 50 mm/min, 100 mm/min, 200 mm/min, 500 mm/min, 750 mm/min and 1,000 mm/min. Samples were tested until break, which occurred near *εr* = 0.45 mm/mm, defined by Equation 4:

(4) $$\varepsilon r=(l-l_{0})/l_{0}$$

where *l* is the extended length of the sample (mm) and *l_0_*, the initial length (200 mm)

#### (c) Cyclic tensile tests

The aim of the cyclic test is to evaluate the effect of repeated elongations on the sensor response. Ten tensile cycles were carried out at a speed of 100 mm/min, between a maximum elongation of 0.04 mm/mm and a minimum of 0 mm/mm. The sample was allowed to relax for 1 s at the maximum deformation, and for 60 s at minimum deformation. The sensor resistance was recorded during the whole test.

### 3.4. Instrumentation of parachute canopy

**Figure 1.**
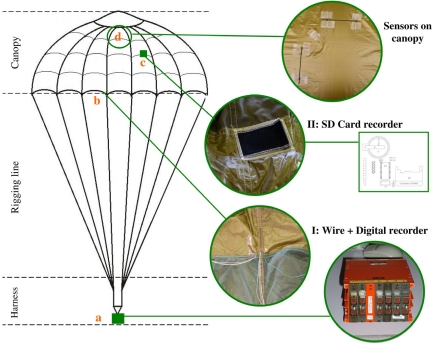
Location of instrumentation on a parachute.

The process described in our previous study [15] and summarized in Section 3.1 was designed so that the sensor could be integrated directly on the parachute canopy without dismantling. To be able to make *in situ* measurement during the inflation phase, it was necessary to develop a specific electronic device to record the sensor data. The first system (I on Figure 1) that has been used was based on a digital recorder (Figure 1a) placed on the harness. However, the integration of the conductive wires in the seam (Figure 1b), from the sensor (Figure 1d) to the recorder, was very difficult. An autonomous recording system (II on Figure 1) based on a Secure Digital Card (SD Card) was therefore developed to overcome this problem. This lightweight miniaturized system is placed in a protective pocket (Figure 1c).

The overall weight of the measurement device is an important element because the parachute canopy is made of a very lightweight fabric. Thus, even a tiny weight placed on the canopy could interfere with the sensor measurement. The overall weight of the “I: Wire + Digital recorder” system is around 270 g (mainly wires) for data recording from four sensors. In comparison, the weight of the alternative “II: SD Card recorder” is close to 60 g (wires, electronics and stitched protective pocket), and able to record data from five sensors simultaneously. The flight test results presented in this paper, were obtained with the initial system.

## 4. Results and Discussion

### 4.1. Thickness of coating

**Figure 2.**
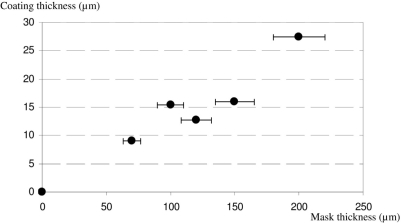
Coating thickness *vs.* mask thickness.

Conductive tracks have been realized using steel masks of different thicknesses ranging from 70 to 200 μm. The thickness of the CPC track plotted against the thickness of the mask is shown in Figure 2. The CPC coating thickness is roughly proportional to the die thickness. It is possible to evaluate its value as approximately equal to 13 % of the mask thickness. This result is close to the theoretical value, since the initial CPC solution was composed of 13.03 vol.−% of solid content (CB + Evoprene) and 86.97 vol. −% of chloroform solvent. This result shows that the amount of CPC absorbed into the nylon fabric is not significant, since the CPC remains preferentially on the fabric surface.

### 4.2. Effect of coating thickness on sensor electrical behaviour

**Figure 3.**
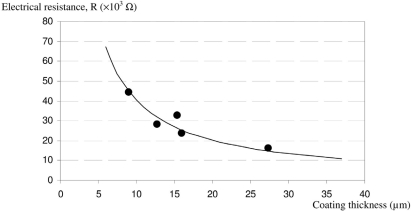
Electrical resistance *vs.* thickness of coated track.

Figure 3 shows the evolution of the electrical resistance of the conductive track with coating thickness. As expected, the electrical resistance decreases when the track thickness increases, in agreement with Equation 1. The data can be modelled by the trend curve on Figure 3 and described by the following expression (Equation 5):

(5) R=0.401/e

The electrical resistivity of the CPC can be calculated from equations 1 and 5: *ρ* *=* 1.26 × 10^−2^ Ω.m.

This value is slightly lower than the usual results obtained for high filler content (>35 wt.−%) based on equivalent CB with different crosslinked matrices (e.g., styrene butadiene rubber [17], chloroprene rubber [18], silicone rubber [19]), or with a thermoplastic matrix (e.g., styrene-ethylene butylene-styrene [20] or poly(ethylene-co-ethyl acrylate) [21]). This behaviour might be due to the excluded volume created by the high amount of CaCO_3_ (30 wt.−% [16]) present in our matrix: CB particles are only confined in the polymer matrix and not in the total volume of the CPC.

#### (a) Aging effect on sensor resistance

**Figure 4.**
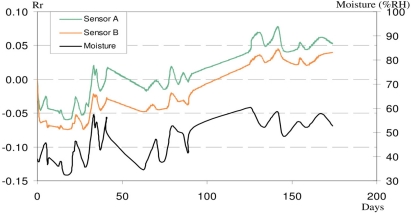
Variation of electrical resistance of sensors *vs.* aging and ambient humidity.

Our previous study [15] showed that the effect of temperature on the sensor resistance was very small in comparison to the effect of moisture. The influence of aging and moisture on the sensor resistance is investigated in this part. Figure 4 shows the relative electrical resistance of two sensors A and B (*Rr*, orange and green lines, left axis) and relative humidity (black line, right axis) plotted over 170 days.

The results show that the electrical resistance of the sensors follows approximately the same trend as the ambient humidity, confirming that this parameter is very influential. The increase in electrical resistance over the 170 days period can therefore be mainly attributed to the increase in relative humidity, and not to an aging effect.

Considering the application of our CPC sensor which was developed for a one shot or occasional measurement, the effect of ambient humidity can be neglected because atmospheric variations of humidity are very slow compared to the duration of a test (less than 5 minutes).

#### (b) Strain rate effect on sensor accuracy

**Figure 5.**
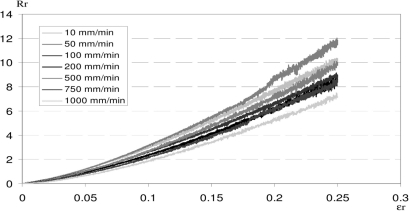
Relative resistance of sensors *vs.* elongation for different strain rates.

Figure 5 shows relative resistance of sensors as a function of elongation for different strain rates, from 10 to 1,000 mm/min. Our CPC sensor can provide data until the break of the fabric (*εr* = 0.45 mm/mm in the weft direction) [15,16], but in this test, sensor performance has been recorded up to 0.25 mm/mm because in the case of parachute canopy inflation, elongations do not exceed 0.20 mm/mm.

**Figure 6.**
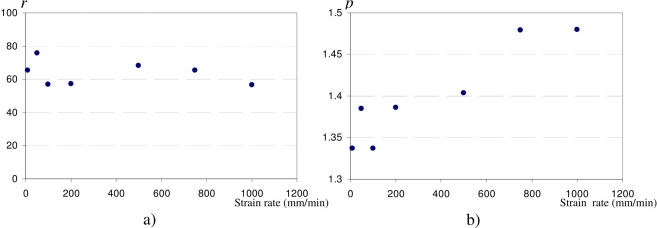
**(a)** Pre-factor *r* and **(b)** exponent *p* vs. strain rate.

Electromechanical behavior does not seem to be significantly affected by the strain rate, since similar curves are obtained for the different test rates. These data can be fitted by a simple power law relation (Equation 6):

(6) $$Rr=r.\varepsilon r^{p}$$

where *Rr*, relative electrical resistance of the sensor (Equation *2*) and *εr*, elongation of the sensor (Equation 4). *r* (pre-factor) and *p* (exponent) are two adjustable parameters. Dependence of these two parameters on strain rate is shown in Figure 6a,b.

Figure 6a shows that the pre-factor (*r*) is independent of strain rate. In fact, this parameter is used to adjust the model to experimental data, and is affected by the geometrical properties of the sensor (dimension and aspect ratio). On the other hand, exponent (*p*) is clearly dependent on strain rate (Figure 6b), as *p* increases with strain rate. This means that sensor sensitivity (*i.e.*, gauge factor) increases when strain rate increases.

The electrical resistance of the CPC is related to the CB conductive networks, which are more or less connected. During elongation, this structure is modified, with more disconnections of the CB networks and thus, an increase in electrical resistance. At a low strain rate, part of the broken conductive networks is balanced by reorganization (slow phenomenon) of part of the conductive particles and polymer matrix chains. At a high strain rate, this compensation is less significant and the sensor response ability increases.

#### (c) Effect of cyclic elongation on sensor accuracy

**Figure 7.**
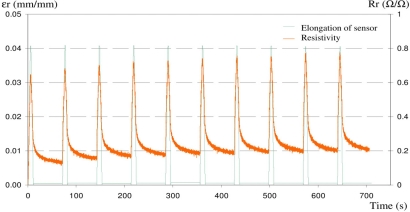
Electrical response of sensor (Rr) during 10 elongation cycles (*εr*).

Figure 7 shows the electrical response (relative resistance *Rr*, orange line) and elongation (*εr*, green line) of sensor during 10 elongation cycles. Maximum elongation was set at 0.04 mm/mm, *i.e.*, within the elastic deformation zone of the fabric.

The sensor is able to detect efficiently all elongation peaks, respecting the elongation cycles quite accurately, but the data show a drift in the electrical response as the number of cycles increases. At minimum deformation (0 mm/mm), *Rr* increases from 0.14 Ω/Ω in the first cycle, to 0.21 Ω/Ω in the last one. In the case of maximum deformation (0.04 mm/mm), *Rr* increases from 0.64 Ω/Ω for the first peak to 0.77 Ω/Ω for the last one. This drift is caused by the long relaxation time of the system (fabric substrate + sensor). After each peak, electrical resistance does not decrease to zero, the time between peaks (60 s) being too short. This kind of behavior, for a carbon based CPC sensor, has already been observed in other studies [22]. Two phenomena can explain this drift (due to relaxation time). The first one is the plastic deformation of the fabric. Even if the strain was kept within the elastic zone of the fabric (*εr* = 0.04 mm/mm), it was observed that after 10 cycles, the total length of the test sample increased by about 1% (*i.e.*, 0.01 mm/mm). This residual length shows that the test sample was plastically stretched. For a woven fabric, this residual length is due to structure reorganization by yarn drifting and rolling and/or plasticization of the yarn’s polymer. Consequently, the sensor on the fabric cannot efficiently measure the total elongation (elastic and plastic). The second explanation is given by the structural organization of the internal CB conductive networks during the cycles. The CB networks are indeed disconnected during elongation, and re-connected during contraction. Theoretically, all broken conductive networks are rebuilt, but in reality, the polymer matrix, mineral fillers and CB nano particles are reorganized during a cycle, and the overall electro-mechanical behavior of the sensor is modified at each cycle. This study confirms the ability of our sensor to detect elongations (especially above *εr* = 0.015 mm/mm). Measurements are nevertheless disrupted by reorganization (elastic and plastic) of the fabric.

### 4.3. Inflation phase measurement

**Figure 8.**
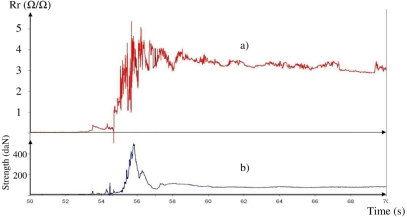
Data recorded by **(a)** CPC sensor on parachute canopy and **(b)** conventional sensor on harness during parachute drop test.

The measurement system called “I: Wire + Digital recorder” was used to record data during a real parachute drop test. The conditions of this first flight test were: weight of dummy: 80 kg; speed: 240 km/h; temperature: 24 °C; Relative Humidity: 65%. In addition to our new CPC sensors placed on the parachute canopy, two conventional sensors were placed on the metallic part of the harness (see localization on Figure 1) and stand as reference. Figure 8 shows data recorded (a) by the CPC sensor, and (b) by the conventional sensor on harness.

The first observation that can be made from this test is that our sensor is perfectly compatible with the conditions of use of a parachute, from the compact packing, flight test and drop, until the landing. In Figure 8(b), it is possible to clearly identify the strong peak (at 55.8 s) corresponding to the inflation shock measured by the conventional sensor on the harness. This peak is also recorded by the CPC sensor on the canopy, but it occurs at a slightly shorter time, less than 1 s, before. The canopy inflates at this moment, and after this the dummy, fastened to the harness, slows down. After the opening shock corresponding to this inflation, the parachute canopy is not submitted to any more strain as it falls, and therefore the CPC sensor resistivity (*Rr*) remains constant (static phase).

The calibration results obtained for the sensor used in this flight test are given by Equation 7 [15]:

(7) $$\varepsilon r={\left(\mathrm{Rr}/\mathrm{1266}\right)}^{\left(\mathrm{1/2.36}\right)}$$

The maximum elongation of the canopy during inflation can be computed from this equation. The peak value of *Rr*, around 4 Ω/Ω, corresponds to an *εr* value of 0.087 mm/mm. This value is slightly higher than results found in literature [3,4,23,24]. However, in these previous studies, measurement was made in a wind tunnel, on a 1/5 scale model and with a constant air flow (*i.e.*, static elongation of canopy). In the present study, the test was made in real conditions of use with a standard parachute.

The measurement system II described in Section 3.4 (Figure 1) was also tested during parachute inflation under real conditions, and was found to be both reliable and able to record data from sensors. However, these data were not available for publication. This lighter system II will therefore be preferred for use in future flight tests.

## 5. Conclusions

This paper follows a previous study on the development of a flexible CPC sensor [15] designed for a textile application, and is focused on the characterization of its electromechanical behavior. Long term measurement has shown that the CPC sensors were not affected by aging and temperature variations (in normal use), but that they were sensitive to ambient humidity. In the case of a one shot atmospheric test, effect of ambient humidity, and its variations, can however be neglected.

Tests with different strain rates (from 10 to 1,000 mm/min) have shown that the global gauge factor was not significantly affected by the strain rate. It seems that one parameter of the power law modeling the electromechanical behavior is slightly dependent on the strain rate. High speed tests (above 1,000 mm/min) have to be done to confirm this tendency.

Cyclic elongation tests have shown that the CPC sensor was able to detect, with good accuracy, elongation peaks of 0.04 mm/mm. Complexity of the mechanical behavior of the fabric substrate and the micro-reorganization of polymer matrix, mineral fillers and CB, caused a drift in the electromechanical measurement. This is probably due to an excessive relaxation time of the system. To further investigate these phenomena, a model fabric substrate with predictable and simple mechanical behavior must be realized.

The use of the CPC sensor in a real flight test has demonstrated that the deformation of a parachute canopy could be successfully measured with this system, especially in the critical inflation phase. This flexible sensor proved to resist the particularly drastic packing conditions of the parachute, the drop, the inflation, and the landing phases. Data from the CPC sensor were coherent with data provided by other conventional sensors and with literature. The results showed that elongation of the fabric during inflation was less than 0.09 mm/mm.
